# Tea polyphenols as an antivirulence compound Disrupt Quorum-Sensing Regulated Pathogenicity of *Pseudomonas aeruginosa*

**DOI:** 10.1038/srep16158

**Published:** 2015-11-09

**Authors:** Honging Yin, Yifeng Deng, Huafu Wang, Wugao Liu, Xiyi Zhuang, Weihua Chu

**Affiliations:** 1 Department of Microbiology, School of Life Science & Technology, China Pharmaceutical University, Nanjing 210009, China; 2 Department of Veterinary Surgery, College of Veterinary Medicine, Nanjing Agricultural University, Nanjing 210095, China; 3 Lishui People’s Hospital, the Sixth Affiliated Hospital of Wenzhou Medical University, Lishui 323000, China

## Abstract

Green tea, a water extract of non-fermented leaves of *Camellia sinensis* L., is one of the nonalcoholic beverages in China. It is becoming increasingly popular worldwide, because of its refreshing, mild stimulant and medicinal properties. Here we examined the quorum sensing inhibitory potentials of tea polyphenols (TP) as antivirulence compounds both *in vitro* and *in vivo*. Biosensor assay data suggested minimum inhibitory concentrations (MICs) of TP against selected pathogens were 6.25 ~ 12.5 mg/mL. At sub-MIC, TP can specifically inhibit the production of violacein in *Chromobacterium violaceum* 12472 with almost 98% reduction at 3.125 mg/mL without affecting its growth rate. Moreover, TP exhibited inhibitory effects on virulence phenotypes regulated by QS in *Pseudomonas aeruginosa*. The total proteolytic activity, elastase, swarming motility and biofilm formation were reduced in a concentration-dependent manner. *In vivo*, TP treatment resulted in the reduction of *P. aeruginosa* pathogenicity in *Caenorhabditis elegans*. When its concentration was 3.125 mg/mL, the survival rate reached 63.3%. In the excision wound infection model, the wound contraction percentage in treatment groups was relatively increased and the colony-forming units (CFU) in the wound area were significantly decreased. These results suggested that TP could be developed as a novel non-antibiotic QS inhibitor without killing the bacteria but as an antivirulence compound to control bacterial infection.

The emergency of finding a solution to multi drug resistance of bacteria due to the abuse of antibiotics leads to the search for new antibacterial pathways. Quorum sensing (QS) or bacterial cell to cell communication is a cell-density dependent bacterial response. It is mediated by hormone-like compounds called autoinducers (AIs). QS-dependent regulation of gene expression controls a wide variety of phenotypes including bioluminescence, biofilm formation, drug resistant, virulence factors expression, and motility. Therefore the inhibition of QS is considered to be a new promising target of antimicrobial pathway as anti-virulence compounds which can repress the gene expression that are essential for basic metabolism *in vitro*, rather than the microorganisms itself^1^,^2^,^3^. *Pseudomonas aeruginosa*, which is responsible for clinical infections, is an opportunist Gram-negative human pathogen, *P. aeruginosa* has three distinct QS systems mediated by the AIs molecules 3-oxo-C12-HSL and C4-HSL, which are produced by the *las* and *rhl* QS systems; and the 4-hydroxy-2-alkylquinolines (HAQs) from the *mvfR* (*pqsR*) QS system^4^. All these three *P. aeruginosa* QS systems are required for the production of virulence factors such as elastase, pyocyanin, exotoxin, biofilm formation, drug resistance and full pathogenicity in mammalian hosts^5^,^6^. Several studies have also indicated the roles of QS in the pathogenesis of *P. aeruginosa* by showing that the QS mutants can cause less tissue damage and decrease mortality rate compared to wild-type *in vivo*^7^,^8^. Disruption of quorum sensing in *P. aeruginosa* has been proposed as a new anti-infective strategy^9^,^10^.

Green tea, a water extract of the non-fermented leaves of *Camellia sinensis L.*, is a very popular beverage in China and is becoming increasingly popular worldwide, partly because of many documented evidences about its beneficial effects on health. It contains numerous components, including catechins, caffeine, amino acids, carbohydrates, proteins, chlorophyll, volatile compounds, fluoride, minerals, and other undefined compounds^11^. Traditional Chinese medicine has considered tea as a medicine and healthful beverage since ancient times. Several biological properties have been associated to tea polyphenols (TP), including antioxidant, anti-carcinogenic and antimicrobial activities. Many studies have shown that the constituents of tea may contribute to human health including the prevention of cancer and cardiovascular diseases, the anti-inflammatory, anti-arthritic, antibacterial, anti-angiogenic, anti-oxidative, antiviral, neuroprotective, and cholesterol-lowering effects^12^. Extract of *Camellia sinensis* can modulate the Quorum sensing of *Pseudomonas aeruginosa*^13^. We hypothesize that some of its antimicrobial properties may be contributed by the QSI phytochemicals present in it.

The purpose of this study is to determine whether QS inhibition activities are present in TP. Furthermore, the influence of TP on *P. aeruginosa* quorum sensing- regulated virulence factors production (protease, elastase, pyocyanin and rhamnolipid), motility and biofilm formation were also assayed. Additionally, we also show the treatment of TP can be a potential candidate to minimize *P. aeruginosa* pathogenesis in *C. elegans*-*P. aeruginosa* and excision wounds mice-*P. aeruginosa* infection model. Our work reveals the importance of TP as a new antivirulence compound against *P. aeruginosa* infection.

## Results

### *In vitro* antimicrobial activity of TP

TP demonstrated powerful antimicrobial activity on all selected microorganisms tested in this work. The MIC of the TP was presented in Table 1. Of the bacteria tested, the MICs of *S. aureus* ATCC 25923 and MRSA were 6.25 mg/mL, 6.25 mg/mL and 12.5 mg/mL, whereas the MIC for gram negative strains as follows: *E. coli* 25922, 6.25 mg/mL, *C. violaceum*12472 and 31532, 6.25 mg/mL, *P. aeruginosa* PAO1, 12.5 mg/mL, Pa1, 12.5 mg/mL, PaR1, 12.5 mg/mL, respectively. To detect the effect of sub-minimum inhibitory concentrations (sub-MIC) of TP on the growth of *C. violaceum* and *P. aeruginosa*, viable cell count methods were used because of the tea polyphenols colour influence the OD600 values. In measuring viable cell counts, 0.5 × MIC of tea polyphenols did not have any antibacterial activity against *P. aeruginosa* Pa1 and *C. violaceum* ATCC12472. A similar result was obtained in the TP treated plates. The results showed that TP concentrations lower than 3.125 mg/mL and 6.25 mg/mL did not have effect on the growth rate of *C. violaceum* 12472 and *P. aeruginosa* Pa1 (data not shown).

### Inhibition of QS-regulated violacein production in *C. violaceum*

**Figure 1:**
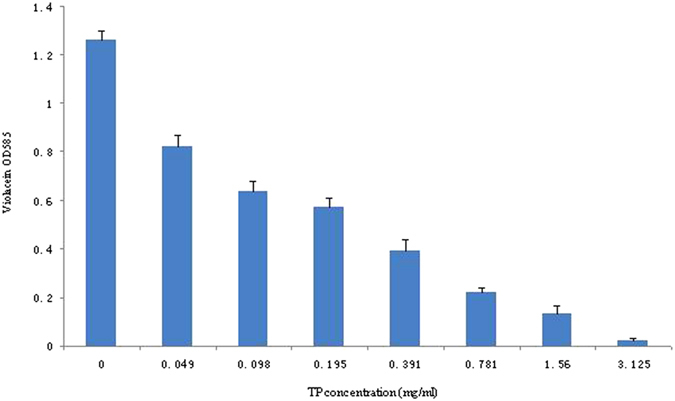
Violacein production by *C. violaceum* 12472 inhibited by different concentration of TP. Values are shown as mean ± standard deviation, n = 3.

The MIC value of TP against *C. violaceum* 12472 was 6.25 mg/mL. We selected 0.5 × MIC of TP (3.125 mg/mL) to further spectrophotometrically measure anti-QS activity of the TP on violacein production of *C. violaceum* 12472. A gradual decrease in the violacein production was observed under treatment with the increasing concentration of TP. Quantitative analysis shows that, TP reduced violacein production dramatically to the level of 82.56% in *C. violaceum* 12472 (at the concentration of 0.781 mg/mL). The inhibition of QS-regulated production of violacein pigment in *C. violaceum* ATCC12472 was in a concentration-dependent manner (Fig. 1).

**Figure 2:**
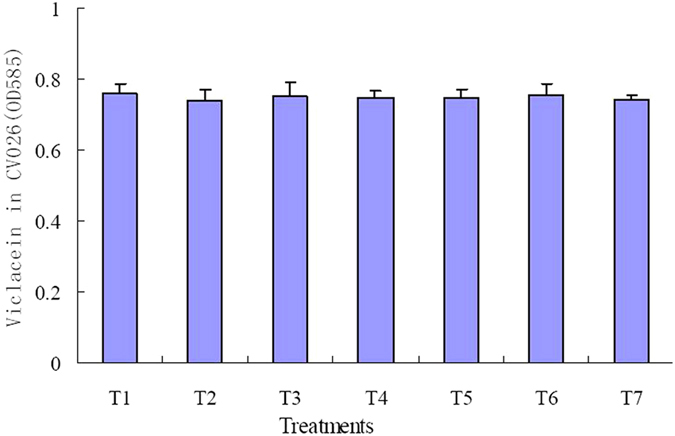
Violacein production by *C. violaceum* CV026 induced by AHL extracted from the culture supernatants of *C. violaceum* 31532 grown in the presence of TP. Values are presented as mean ± SD, n = 3.

**Figure 3:**
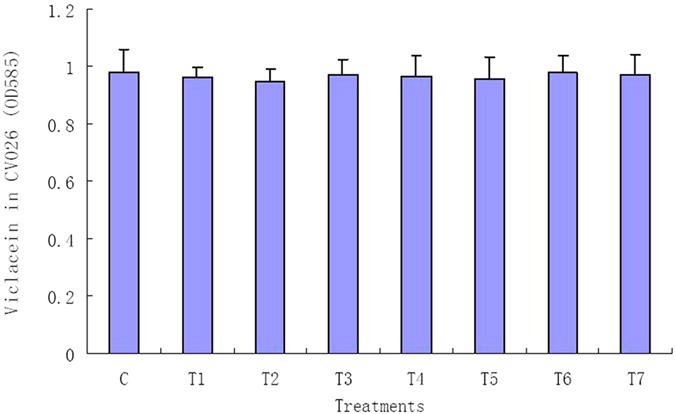
Violacein production by *C. violaceum* CV026 induced by C6-HSL treated with TP. Values are presented as mean ± SD, n = 3.

The effects of TP on AHL synthesis and its activity were determined. The AHL extracted from the culture supernatants of CV31532 in the presence of TP was able to induce violacein production in *C. violaceum* CV026, and there is no significant difference among different concentration of TP (Fig. 2). Activity of C6-HSL was not decreased after incubated with TP (Fig. 3). These results indicate that TP will not affect AHL synthesis and its activity.

### Inhibition of swarming motility in *P. aeruginosa* Pa1 by TP

**Figure 4:**
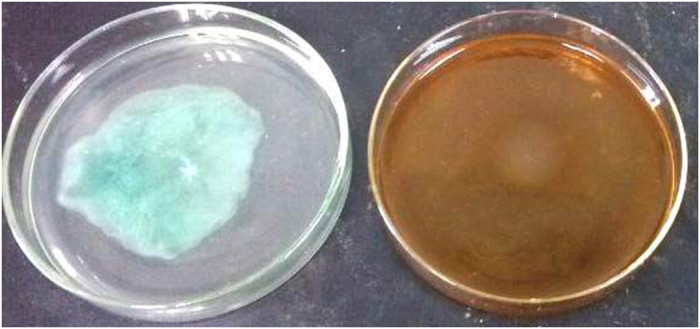
Inhibition of swarming motility on LB medium with 0.3% agar. Shown are swarming Pa1 on agar plates with TP (0 mg/mL) (**A**) and (50 μg/mL) (**B**) respectively.

Inhibition of swarming motility in *P. aeruginosa* Pa1 was observed at concentrations as low as 0.05 mg/mL TP (Fig. 4). *P. aeruginosa* Pa1 exhibited swarming motility on LB agar plates at the point of inoculation with a total swarming diameter of 60 mm. In the presence of TP, the bacteria were able to grow and form a colony in the center with a diameter not exceeding 10 mm, and tendril formation or other features indicating of swarming motility were not observed.

### TP decreased the production of QS-regulated virulence factors in *P. aeruginosa* Pa1

**Figure 5:**
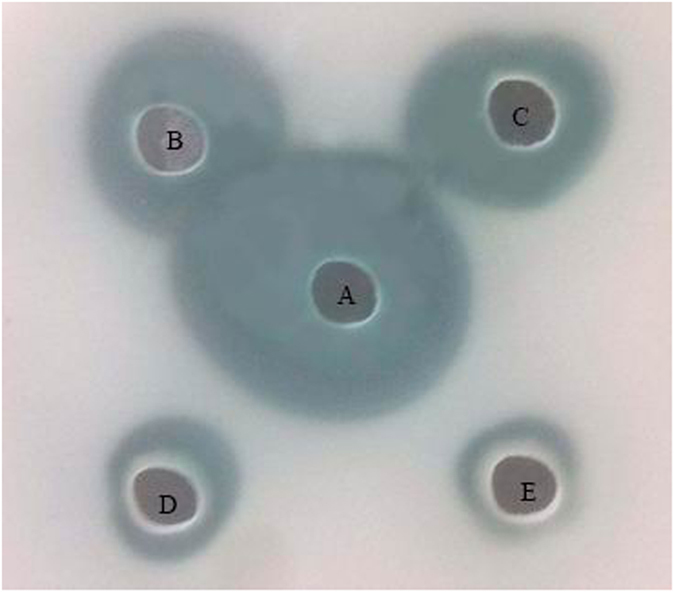
Effect of TP on the protease activity. The total proteolytic activity of the culture supernatant of *P. aeruginosa* Pa1 treated with TP on skim milk plate. (**A**) PBS control, (**B**) 0.049 mg/mL, (**C**) 0.159 mg/mL, (**D**) 0.781 mg/mL, (**E**) 3.125 mg/mL.

**Figure 6:**
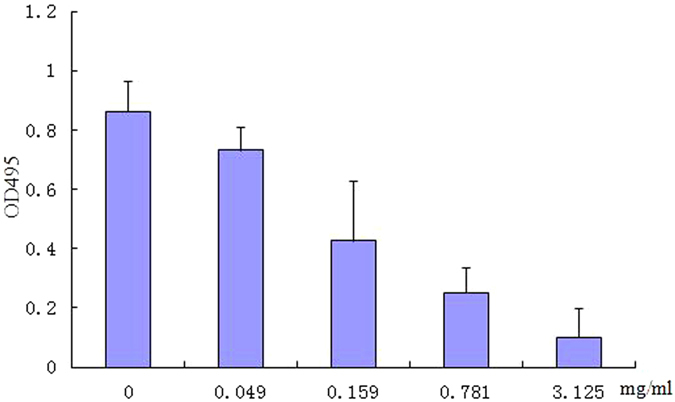
Effect of TP on elastase activity in *Pseudomonas aeruginosa* Pa1 (a). Values are presented as mean ± SD, n = 3.

The ability of sub-MIC TP in reducing QS-dependent protease and elastin-degrading elastase activity was assessed. As shown in Fig. 5, TP clearly decreased the protease activity in the supernatant of TP-treated Pa1, with that of untreated Pa1 supernatant. TP can significantly inhibit elastolytic activities at 0.049–3.125 mg/mL concentrations (Fig. 6). At 0.049 mg/mL, 15.1% inhibition of elastolytic activities was observed, and almost 88.3% inhibition of elastolytic activities was evident at 3.125 mg/mL TP.

**Figure 7:**
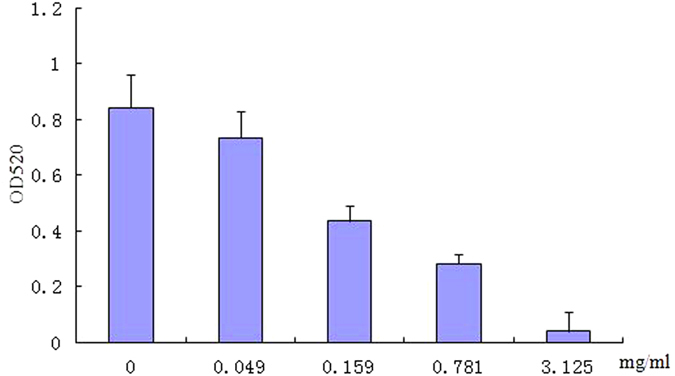
Effect of TP on pyocyanin production in *Pseudomonas aeruginosa* Pa1 (a). Values are presented as mean ± SD, n = 3.

Different concentrations of TP were tested on pyocyanin production in *P. aeruginosa* Pa1. As shown in Fig. 7, TP concentrations below MIC had a significant impact on pyocyanin production without affecting *P. aeruginosa* Pa1 growth. At 0.049 mg/mL, there was a 12.7% decrease in pyocyanin production and at 3.125 mg/mL, almost complete inhibition of pyocyanin production was observed.

**Figure 8:**
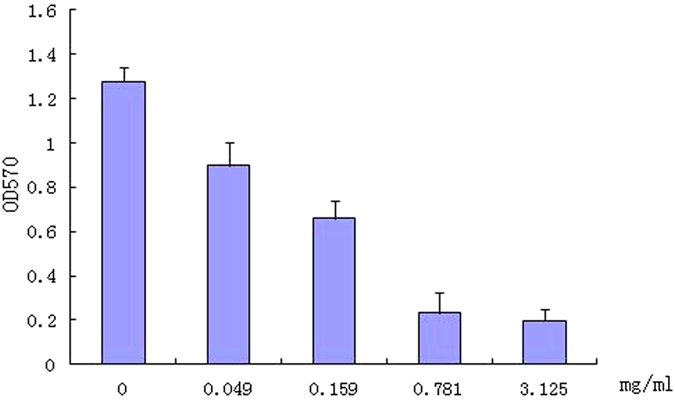
Effect of TP on biofilm formation in *Pseudomonas aeruginosa* Pa1 (a). Values are presented as mean ± SD, n = 3.

Biofilm formation is partially controlled by QS mechanisms. Therefore, the effect of TP on biofilm formation in *P. aeruginosa* Pa1 was assessed after 24 hours growth and interestingly, at 3.125 mg/mL TP, the biofilm formation was decreased by >80% (Fig. 8).

### Exogenous Supplementation of TP Prevents Pa1 Killing of *C. elegans*

**Figure 9:**
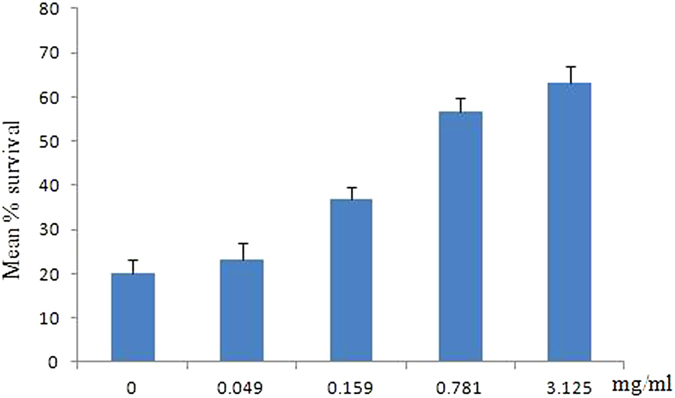
Effect of TP on the survival rate of *C. elegans* inflicted by *P. aeruginosa* Pa1. TP supplementation in the medium reduced Pa1 pathogenicity, resulting in reduced mortality of *C. elegans*. Results are the means of three independent experiments, and standard deviations are shown

To determine whether TP can decrease pathogenicity of *P. aeruginosa*, we used *Caenorhabditis elegans* killing infection assay. We found that TP can increase the survival rate of *Caenorhabditis elegans* when infected with *P. aeruginosa* Pa1. Figure 9 shows the percentage survival of worms after 48 h exposure to Pa1 with different sub-MIC concentration of TP. Only about 20% of Pa1-infected worms survived, whereas worms exposed to *Escherichia coli* OP50 remained alive throughout the assay (data not shown). In comparison, treatment of infected worms with TP at sub-MIC concentrations can significantly improve its survival (23.3–63.3%). The highest survival rate (63.3%) was obtained with 3.125 mg/mL TP.

### Effect of TP on *P. aeruginosa* -mouse infection model

Finally, *P. aeruginosa*-mouse infection model was used to investigate potential beneficial effects of TP *in vivo*. The wound skin and infection assays were performed. Skin wounds were made on the back side of mouse and bacterial infection was initiated by dropping 1 × 10^7^CFU of *P. aeruginosa*. Then the infected wound skin sites were pipetted with either different concentrations of TP or sterilized PBS. After 6, 9, 12 and 15 days post-infection, the number of recoverable bacteria from the wounds area were determined. The number of CFU from the wounds after infection of *P. aeruginosa* was detected in all groups. There was no statistically significant difference between these groups on the first day post infection, but the bacterial number in wound area of TP-treat group decreased significantly on day 3 and 6 post-infection compared to control group (Table 2). In the process of wound healing, bacterial infection to skin wounds led to a significant delay in the closure of excisional wound sites compared with non-infected wound sites. When TP used to treat the infected wound skin sites, however, wound healing process was dramatically accelerated. The area of wound was measured on the 6th, 9th, 12th and 15th day of post infection in all groups. A very high significant rate of closure of wound was observed between 9th and 15th day post surgery (*P* < 0.05). The percentage of wound healing of the extracts against the post infection days was presented in Table 3.

## Discussion

*Pseudomonas aeruginosa*, a common pathogen, which is important in cystic fibrosis, burn units of hospitals, and in implanted medical devices including intubation tubes and stents. Inhibition of the QS systems is considered as a novel strategy for development of potential anti-infective therapy. In the current study, we have demonstrated that TP treatment of the clinical wound infected isolated strain Pa1 can both attenuate biofilm formation and down-regulate the production of extracellular virulence factors *in vitro*. TP-treated Pa1 also exhibited reduced virulence in both *C. elegans* and mice infection models.

Green tea extracted mainly consists of 4 kinds of catechins namely (−)-epicatechin (EC), (−)-epicatechin gallate (ECG), (−)-epigallocatechin (EGC), and (−)-epigallocatechin gallate (EGCG)^13^. Green tea polyphenols have a wide spectrum of activity against different pathogenic bacteria and also drug resistant bacteria, including strains of *P. aeruginosa.* Our MIC data were similar to those obtained by others^14^,^15^. Some medical plant extracts which have antibacterial activity with higher minimum inhibitory concentration values have demonstrated anti-QS activity even at lower concentrations^16^. This study clearly demonstrates that TP at sub-MIC has the ability to counter the QS system. We found that the growth of *C. violaceum* was not affected at sub-MIC of TP. TP inhibited almost 98% reduction in violacein pigment production when at 3.125 mg/mL. This result corroborates well with the finding of others. *Rosa rugosa* tea polyphenol (RTP) extract inhibited QS-controlled violacein production in *C. violaceum* 026 with 87.56% reduction without significantly affecting its growth at the concentration of 1.20 mg/ml^17^. Yang’s results suggested that EGCG has a higher binding affinity towards the enoyl-acyl carrier protein reductase of *P. aeruginosa* and is an efficient quorum-quenching reagent^18^. Vattem *et al.* found that the inhibition of violacein production in *C. violaceum* 026 by certain spices containing high concentrations of phenolic compounds to the level of about 41%^19^. Inhibition of QS system can be achieved either by interruption of AHL signal molecules synthesis, inhibition of AHL signal dissemination or inhibition of AHL to conjugate with the signal receptor^20^. Our results indicated that TP inhibit the QS system of *C. violaceum* was not by the production of AHLs and degrading AHLs, it maybe interfere with the AHLs receptors. We found that TP can inhibit the production of total protease, elastase, pyocyanin, biofilm formation and swarming motility in *P. aeruginosa* without inhibiting its growth. Compared with previous studies, phenolic compounds of ginger and their derivatives displayed significant QS inhibitory effects. They can significantly decrease the production of pyocyanin to the level of 83–90%^21^. Epigallocatechin gallate (EGCG), one of the compounds of tea polyphenols can inhibit the formation of biofilms by 30% reduced at a concentration of 40 μg/mL. Besides biofilm formation, 40 μg/mL EGCG significantly reduced the swarming ability of *Burkholderia cepacia*^22^. All these studies strongly suggest that polyphenols’ anti-virulence effect is due to QS inhibition.

However, since effects obtained from *in vitro* model systems cannot always be reproduced under *in vivo* conditions. For this reason, we used *C. elegans*-*P. aeruginosa* and excision wounds mice-*P. aeruginosa* infection model to investigate the anti-virulence ability of TP. The susceptibility of *C. elegans* with different virulent phenotypes of *P. aeruginosa* makes the worms an excellent model for studying host–pathogen interactions^23^. We observed a considerable reduction in the killing rate of *C. elegans* following sub-MIC TP-treatment groups compared with control. The highest survival (63.3%) was obtained with 3.125 mg/mL TP. These observations are in agreement with previous reports on other phenolic compounds. Curcumin, a known phenolic from *Curcuma longa*, attenuates the virulence of *P. aeruginosa* PAO1, with increasing concentrations of exogenously supplemented curcumin^24^. *C. elegans* N2 treated with whole apple extracts (polyphenols) showed increased resistance to the pathogen *P. aeruginosa*^25^.

Wound healing is caused by complex, multifactor effects involving several cellular and biochemical processes. In the process of wound healing, bacterial infection to skin wounds led to a significant delay in the closure of excisional wound sites, so anti-infection is important in wound healing. In this study, we found that the number of recoverable CFU from the wounds after infection of *P. aeruginosa* Pa1 was not significantly different between TP treated groups and the control group on the first day post infection, but the bacterial number was significant decreased in wound area on day 3 and 6 post-infection in TP treated groups compared to control group. Our results are similar to Gupta *et al.*^26^. Their study showed that topical application of lactonase enzyme reduced the systemic spread of *P. aeruginosa* in thermal injury infection mice model as depicted by lower bacterial counts in skin and blood. This may be result from quenching the QS signal molecules which leads to inhibit the production of virulence factors, and finally delayed its infection. Wound size reduction treated with tea polyphenols dramatically accelerated as shown in Table 2. Kapoor *et al.* demonstrated that epicatechin gallate (ECG), one of the catechin components in green tea, could significantly improve the quality of wound healing and scar formation in an incisional wound healing model in rats^27^. Klass *et al.* results also indicated that EGCG has potential effects on wound contraction and healing^28^. The results of the present study shown that tea polyphenols at sub-MIC can promote wound healing, along with other information about green tea in the literature, strongly suggests that tea polyphenols may be beneficial in wound healing by its antioxidant and anti-inflammatory ability and may contribute in recovery of burn wounds and scars^29^,^30^.

In the present study, we highlighted that TP can inhibit the production of QS-regulated virulence factors and biofilm formation. TP also helps to attenuate the virulence of *P. aeruginosa in vivo*, which resulted in reduced pathogenicity of this pathogen in both *C. elegans* and mice infection models. *In vitro* attenuation of virulence factors correlated well with the *in vivo* study. These observations suggest that TP has the prophylactic potential to be a new antivirulence compound against *P. aeruginosa* infection.

## Materials and Methods

### Materials

Tea polyphenols (TP) extracted from the leaf of *Camellia sinensis* L. were purchased from Zhejiang University Tea Scientific Co., Ltd (purity >98%, Hangzhou, China). HPLC chromatographic analysis (data obtained from the manufactory) TP containing 78% total catechins, and the catechins contained five main compounds: ~48% EGCG, ~26% ECG, ~15% GCG, ~2% EC, and ~1% EGC. Stock solutions were prepared by dissolving 1g of powder in 1mL of 0.15 mM H_3_PO_4_ to avoid oxidation and filtering the solution through a 0.22-μm-pore-size membrane filter. The stock solution was stored at −20 °C until use.

### Strains and culture conditions

*Escherichia coli* (ATCC 25922 and OP50), *Staphylococcus aureus* (ATCC 25923), *Chromobacterium violaceum* (ATCC12472, ATCC31532 and CV026), clinically isolated methicillin resistant *S. aureus* (MRSA-1 and MRSA-2), *Pseudomonas aeruginosa* PAO1 (ATCC 27853), clinically isolated stains Pa1 (isolated from wound infection) and multidrug resistant strain PaR1 were used in this study. The tested microbial strains were provided by Department of Microbiology, School of Life Science & Technology, China Phamarceutical University. Each isolate was maintained on a Luria Bertani (LB) slant at 4 °C and activated at 37 °C for 24 h with a LB plate prior to any antimicrobial and QS inhibitory tests.

*Caenorhabditis elegans* N2 (Bristol) was propagated under standard conditions, synchronized by hypochlorite bleaching, and cultured on nematode growth medium at 20 °C using *E. coli* OP50 as standard food source^31^.

### Determination of minimum inhibitory concentration (MIC)

Tea polyphenols were tested against the selected bacteria strains for their inhibitory activity using a modified broth micro-dilution method according to Clinical and Laboratory Standard Institute^32^. Briefly, serial two-fold dilutions (100–0.781 mg/mL) of tea polyphenols were prepared in MHB, at a volume of 100 μl per well in 96-well U-bottom micro-titer plates (Nunc, Denmark). Each well was inoculated with 5 μl of the standardized inoculum, corresponding to a final test concentration of about 1–5 × 10^5^ CFU/mL. After incubation at 37 °C for 24 h, the MIC was calculated as the lowest concentration of the TP that completely inhibited visible growth. The sub-MIC concentration was selected for the assessment of anti-biofilm and anti-QS activity.

### Effect of tea polyphenols on bacterial growth

Effects of TP on bacterial growth were determined by measuring cultures’ CFUs when treated with TP at sub-MICs. CFUs were measured by counting colonies after plating 1 mL of each culture on LB plates and incubating the plates overnight and then count the colonies.

### Quantitative QS inhibition assay

The effect of TP on the QS-controlled violacein production in *C. violaceum* ATCC12472 was determined as follows. Briefly, 5 mL LB broth containing different concentrations of tea polyphenols was inoculated with 100 μL *C. violaceum* ATCC12472 (10^6^ CFU/mL). All the tubes were incubated at 30 °C for 24 hours in an orbital shaking incubator (150 rpm). Violacein was extracted by water-saturated butanol according to the method of Blosser and Gray^33^ and was quantified spectrophotometrically at optical density (OD) 585 (UV-1800; Shimadzu).

### Effect of TP on modulation of AHL synthesis and its activity

The effect of TP on AHL synthesis was determined as described by Vattem *et al.*^19^. *C. violaceum* ATCC31532 was cultured in the presence of TP at a concentration of 0.049–3.125 mg/mL for 24 h incubation. AHL was extracted from the cell free supernatant (5 mL) using dichloromethane (3:1 v/v) and evaporated under a thin stream of nitrogen gas. For determining the AHL activity, the dried AHL fractions were re-suspended in 70% methanol (20 μL) and added to fresh 10 mL LB medium inoculated with biosensor strain *C. violaceum* CV026 which responded to exogenous AHL by producing violacein. Induction of violacein by the AHL fractions in *C. violaceum* CV026 was measured spectrophotometrically after incubation at 30 °C for 24 h. For the detection of TP on AHL activity, aliquots of C6-HSL (10 μg/μl) (Sigma-Aldrich, St. Louis, Missouri, USA) in absolute ethanol were dispensed into sterile tubes and the solvent evaporated to dryness under sterile condition. TP was added to the tube to rehydrate the C6-HSL to a final concentration of 0.1 μg/μl. The mixtures were incubated at 37 °C for 6 h with gentle shaking in a hybridization oven and then the AHL activity was detected.

### QS inhibitory efficiency of TP against QS controlled virulence factors of *P. aeruginosa*

#### Swarming motility inhibition assay

Five milliliters of molten soft top agar (0.3 g agar, 1.0 g tryptone, 0.5 g Yeast extract powder, 0.5 g sodium chloride, 100 mL deionized water) with the final concentrations of TP were 50 μg/mL. Then, poured it immediately over the surface of a solidified LB agar plate as an overlay and allowed to dry for 3 h at 30 °C. The plates were then center point inoculated with Pa1 and incubated at 37 °C for 24 h. The extent of swarming was determined by measuring the diameter of the motility swarms^34^.

#### Virulence factor assays of *Pseudomonas aeruginosa*

Overnight cultures of Pa1 were diluted 1:100 after growth in LB medium and incubated with different concentration of TP or PBS as a control. After growth for 24 h at 37 °C, the culture was then centrifuged (8000 rpm, 4 °C, 10 min). Then the virulence factors were determined in cell-free supernatant fluid aliquots. As *P. aeruginosa* produces diverse virulence factors, we only assayed the following four phenotypes.

Total proteolytic activity of the culture supernatant was determinated according to the method described by earlier^35^. Briefly, skim milk agar plates containing 10 g of nonfat dry milk (skim milk) and 1 g of agar in 100 mL of distilled water. Holes were made on the milk agar plate. Culture supernatants (50 uL) of *P. aeruginosa* were added in a hole and incubated at 37 °C for 24 h. Proteolytic activity was measured by the diameter of the clear zone surrounding the holes.

Elastase activity was measured by modifying the methods previously described^36^. Briefly, to 100 mL of culture supernatant, 900 ul of elastin-congo red (ECR) buffer (100 mM Tris, 1 mM CaCl2, pH 7.5) containing 20 mg ECR (Sigma Chemical Co., [St. Louis, MO]) was added and then incubated at 37 °C for 3 h in a water bath. The samples were then centrifuged (1500 rpm for 10 min at 4 °C) to remove insoluble elastin-congo red. The absorbance of the supernatant from both the control and treated samples was determined by reading OD495. The percent change in absorbance was then calculated to determine the decrease in elastase activity.

Pyocyanin was extracted from culture supernatant fluids and quantification assay was performed as previous described with slight modification^37^. Briefly, 3 mL of chloroform was mixed with 5 mL culture supernatant fluid. The chloroform layer was transferred to a fresh tube and mixed with 1 mL 0.2 M HCl. After centrifugation, the top layer (0.2 M HCl) was removed and its absorption measured at 520 nm.

Quantification of biofilm was based on tube method described by Christensen *et al.*^38^. 100 uL overnight cultures were added to 5ml LB with different concentration of TP. Uninoculated LB tubes were used as negative controls. The tubes were incubated for 24 hours at 37 °C. After incubation, tubes were decanted and washed with phosphate buffer saline (pH 7.3) and dried. Tubes were then stained with 0.1% crystal violet for 5 minutes. Excess stain was rinsed off by deionized water. After that the tubes were air dried, the dye bound to the adherent cells was resolubilized with 33% (v/v) glacial acetic acid The Optical density (OD) was measured at 570 nm using Spectrophotometer (UV-1800 Shimadzu, Japan). The test was made in triplicates and repeated three times, and the data was then averaged.

### *In vivo* models for TP as non-antibiotic agents for *P. aeruginosa* infection control

#### Effect of TP on the pathogenicity of *P. aeruginosa* Pa1 in *Caenorhabditis elegans*

Pseudomonas Infection Agar (PIA) was prepared in 60 mm plates as described by Tan^39^. After pour the plates, while the medium were still in liquid form, TP was added to the plates and mixed thoroughly, and the plates with different concentration below MIC of TP were prepared. For killing assay, 10 ul of an overnight Pa1 culture was evenly spread onto the assay plates. The plates were then incubated at 37 °C for 24 h and allowed to reach room temperature before seeding with 30 young adult worms and conducted in triplicates. Percentage survival of the infected worm population was determined after further 48 h incubation at 20 °C.

#### Mouse model and wound healing assay

Excision wound models were used to evaluate the wound healing activity of TP against *P. aeruginosa* infection. Animal infection and wound healing experiments were performed as previously described by Mughrabi *et al.*^40^ with minor modification. 7–8 weeks healthy Kunming male mice weighing (20 ± 2) g purchased from the Experimental Animal Center of Southeast University, China were used for the experiment. All studies were performed in compliance with the National Institutes of Health Guide for the Care and Use of Laboratory Animals and approved by IACUC (Institutional Animal Care and Use Committee of China Pharmaceutical University). The mice were anesthetized with an intraperitoneal (IP) injection of pentobarbital sodium (0.05 mg/g weight), and the dorsum was shaved by razor and disinfected with 75% ethanol. A full thickness excision (2 × 2 cm) was made at the impressed area. The mice were then infected with a clinical strain of *P. aeruginosa* (Pa1) by pipetting the organisms directly into the wound (~1 × 10^7^ CFU/wound). Eight mice were used for each group and three groups were utilized. Group A received PBS as control; Group B received 200 ul low concentration TP (0.098 mg/mL); Group C received 200 uL high concentration TP (3.125 mg/mL), and then covered with plain gauze. It was applied topically once daily by pipetting the solutions directly into the wound area. The animals were then placed into individual cages. The measurements of the wound areas of the excision wound model were taken on 6th, 9th, 12th and 15th day following the initial wound using transparent paper and a permanent marker. The recorded wound areas were measured with graph paper. The percent wound contraction was calculated using the following equation: Wound Contraction (%) = [Initial wound size − Specific day wound size]/Initial wound size × 100^41^.

Bacteria in the wound area were also detected on 1st, 3rd, and 6th day post infection. Swabs were taken from the wound, and the collected swabs were used to detect the bacterial counts in the wound. Briefly, 1 mL of normal saline was added to each of the samples. The sample was vortexed thoroughly and a 10-fold serial dilution was performed. One hundred microliters of each sample dilution was spread onto LB agar plate. Two replicates were carried out for each dilution, and the agar plates were incubated at 37 °C for 24 hours. The colonies were counted, and results were tabulated^42^.

### Statistical analysis

The values were expressed as means ± standard deviations (SD) of at least three assays. Analysis of variance was conducted and differences between variables were tested for significance by one-way ANOVA with T-test using the SPSS V16.0 program. Differences at *P* <  0.05 were considered statistically significant.

## Additional Information

**How to cite this article**: Yin, H. *et al.* Tea polyphenols as an antivirulence compound Disrupt Quorum-Sensing Regulated Pathogenicity of *Pseudomonas aeruginosa*. *Sci. Rep.* **5**, 16158; doi: 10.1038/srep16158 (2015).

## Figures and Tables

**Table 1:** Minimum inhibitory concentrations of TP against selected microorganisms.

TP concentration (mg/mL)	Minimum inhibitory concentration								
	*S. aureus*			*E. coli*25922	*C. violaceum*		*P. aeruginosa*		
	25923	MRSA1	MRSA2		12472	31532	PAO1	Pa1	PaR1
100	−	−		−	−	−	−	−	−
50	−	−		−	−	−	−	−	−
25	−	−		−	−	−	−	−	−
12.5	−	−	−	−	−	−	−	−	−
6.25	−	−	+	−	−	−	+	+	+
3.125	+	+	+	+	+	+	+	+	+
1.562	+	+	+	+	+	+	+	+	+
0.781	+	+	+	+	+	+	+	+	+

**Table 2:** Bacterial load of wound area post *P. aeruginosa* infection.

Animal groups	Treatment	Lg bacteria count/cfu		
		d 1	d 3	d 6
A	PBS + Pa1 infected	6.24 ± 0.67	7.2 ± 0.69	7.4 ± 0.63
B	TP low dose, + Pa1 infected	6.08 ± 0.54	5.67 ± 0.60	4.63 ± 0.49
C	TP high dose, + Pa1 infected	5.99 ± 0.72	5.43 ± 0.83	4.42 ± 0.35

**Table 3:** Effect of TP on the wound healing in wound dripped with *P. aeruginosa.*

Animal groups	Treatment	Rate of wound healing %			
		d 6	d 9	d 12	d 15
A	PBS + Pa1 infected	19.2 ± 6.5	40.2 ± 6.0	55.6 ± 8.2	76.1 ± 4.9
B	TP low dose, + Pa1 infected	21.3 ± 5.4	52.6 ± 4.7^*^	68.9 ± 5.9^*^	88.9 ± 2.9^*^
C	TP high dose, + Pa1 infected	29.7 ± 7.3^*^	60.1 ± 6.6^*^	74.9 ± 5.8^*^	92.1 ± 2.3^*^

Results are expressed as mean ± SEM. (n = 8). *P* < 0.05 when compared to control group (one-way ANOVA followed by t-test).
