# Corrigendum: Tea polyphenols as an antivirulence compound Disrupt Quorum-Sensing Regulated Pathogenicity of *Pseudomonas aeruginosa*

**DOI:** 10.1038/srep17987

**Published:** 2016-01-12

**Authors:** Hongping Yin, Yifeng Deng, Huafu Wang, Wugao Liu, Xiyi Zhuang, Weihua Chu

Scientific Reports 5: Article number: 1615810.1038/srep16158; published online 11092015; updated on 01122016

The original version of this Article contained a typographical error in the spelling of the author Hongping Yin, which was incorrectly given as Honging Yin. This has now been corrected in the PDF and HTML versions of the Article.

